# An Intensified Regimen Containing Linezolid Could Improve Treatment Response in *Mycobacterium abscessus* Lung Disease

**DOI:** 10.1155/2019/8631563

**Published:** 2019-11-19

**Authors:** Hua Li, Li Tong, Jun Wang, Qingtao Liang, Yun Zhang, Naihui Chu, Xiaoyou Chen, Hongfei Duan

**Affiliations:** Department of Tuberculosis, Beijing Chest Hospital Affiliated to Capital Medical University, Beijing Tuberculosis & Thoracic Tumor Research Institute, Beijing, China

## Abstract

**Background:**

Treatment response for the *Mycobacterium abscessus* (*M. abscessus*) lung disease remains far from satisfying. An effective regimen is needed to solve the problem.

**Methods:**

We retrospectively reviewed the medical records of all patients with *M. abscessus* lung disease who received antibiotics regimen at Beijing Chest Hospital Affiliated to Capital Medical University between July 1, 2010, and February 1, 2018. Patients were administered a conventional antibiotics regimen (including macrolide and moxifloxacin, along with an initial 12-week course of low-dose cefoxitin and amikacin) or intensified regimen (including a higher dosage of cefoxitin and linezolid besides conventional drugs), respectively. The time to sputum-culture conversion and proportion of sputum-culture conversion in liquid broth were investigated to evaluate the efficacy and evaluation of safety by performing the classification of adverse events according to the Division of AIDS, National Institute of Allergy and Infectious Disease. Patients were followed for 18 months from baseline.

**Results:**

In the conventional regimen group, the sputum conversion rate at 18 months was 29.4% (10/34), and the median time until sputum conversion was 2 months (IQR, 1-2 mo). Furthermore, in the intensified regimen group, the sputum conversion rate was 81.3% (13/16), and the median time until sputum conversion was 1 month (IQR, 1-1 mo). Leukopenia and drug-induced hepatotoxicity occurred more frequently in the intensified regimen group in contrast with the conventional regimen group patients. However, only 1 adverse event in the intensified regimen group was classified as severe adverse event.

**Conclusions:**

The intensified regimen could improve sputum conversion of *M. abscessus* lung disease compared with conventional regimen, but close safety surveillance is necessary to monitor adverse events.

## 1. Introduction

Despite significantly variable geographic distributions, *Mycobacterium abscessus* (*M. abscessus*) complex is one of the most important pathogens responsible for causing pulmonary nontuberculous mycobacteria (NTM) diseases worldwide [1]. In some East Asian countries, *M. abscessus* complex is the second most common pathogen responsible for NTM lung diseases after the *Mycobacterium avium* complex (MAC) [2, 3].

Among the *M. abscessus* complex lung diseases, *M. abscessus* lung disease is more refractory to antibiotics in contrast with the *Mycobacterium bolletii* and *Mycobacterium massiliense* lung diseases [3–6]. Furthermore, a standard treatment regimen, recommended by guidelines, remains unavailable. Usually, a combinatory regimen including cefoxitin, fluoroquinolone, macrolide, and amikacin is used to treat *M. abscessus* disease [7]. Although culture conversion for the *M. abscessus* lung diseases reached ∼60% in the previous report in Korea, frequent adverse reactions due to antibiotics limit usage of the regimen [7]. The aim of this study was to compare the efficacy and safety of an intensified regimen containing linezolid and a conventional regimen.

## 2. Methods

### 2.1. Study Population

We retrospectively reviewed the medical records of all patients with *M. abscessus* lung disease who received antibiotics regimen at Beijing Chest Hospital Affiliated to the Capital Medical University between July 1, 2010, and February 1, 2018. Patients with *Mycobacterium bolletii* and *Mycobacterium massiliense* lung diseases are excluded from the study. According to the risk for the participants and their information, the study was authorized the waiver for the informed consent by the hospital IRB.

### 2.2. Diagnosis of *M. abscessus* Lung Disease

Mycobacterial cultures were prepared as recommended in the standard guidelines. All cultures were grown in the BACTEC MGIT 960 system. NTM species were identified by gene sequence analysis of the 16S ribosomal RNA, *rpoB* and *hsp65* genes, as previously described [8].

The computed tomographic images obtained at the time of diagnosis of *M. abscessus* complex lung disease were reviewed as the baseline CT in terms of disease patterns (i.e., either nodular bronchiectatic or fibrocavitary type).

Patients were diagnosed with *M. abscessus* lung diseases based on the diagnostic criteria of the American Thoracic Society (ATS)/Infectious Diseases Society of America (IDSA) guidelines. All patients met clinical, microbiological, and radiographic criteria [1].

### 2.3. Treatment and Response Assessment

From July 1, 2010, to July 31, 2017, patients commencing antibiotic therapy were administered macrolide (clarithromycin 1,000 mg/d or azithromycin 250 mg/d) and moxifloxacin (400 mg/d), along with an initial 12-week course of cefoxitin (4 g/d in two divided dose) and amikacin (400 mg/d for weight ≤ 50 kg, 600 mg/d for weight > 50 kg).

From Aug 1, 2017, to Feb 1, 2018, patients were administered azithromycin (250 mg/d), moxifloxacin (400 mg/d), and linezolid (600 mg/d for the first 3 months, followed by 300 mg/d for the following months) along with an initial 4-week course of cefoxitin (12 g/d in three divided dose for weight ≤ 50 kg or 8 g/d in two divided dose weight > 50 kg) and 12-week amikacin (400 mg/d for weight ≤ 50 kg, 600 mg/d for weight > 50 kg). Both the conventional regimen and intensified regimen last 12 months after the first sputum conversion. Patients were followed for 18 months from baseline.

Complete blood cell counts, serum creatinine, and liver function test results were monitored twice a week in the first month. Imipenem (500 mg, three times a day) was used as a substitute if an adverse reaction associated with cefoxitin occurred.

### 2.4. Assessment of Microbiologic Response

Sputum-culture examinations were performed monthly until the end of treatment. Sputum conversion was defined as three consecutive negative cultures, taken at least 28 days apart, and found to be negative, with the time of conversion defined as the date of the first negative culture [9]. In such cases, the date of collection of specimen from the first negative culture is used as the date of conversion. If the patient could not expectorate sputum during treatment, the sputum was considered to have converted. Sputum reversion was defined as after an initial conversion, two consecutive cultures, taken at least 28 days apart, are found to be positive [10].

### 2.5. Assessment of Adverse Events

Adverse events were classified according to the Division of AIDS, National Institute of Allergy and Infectious Disease, and were recorded at any time during the treatment ([Supplementary-material supplementary-material-1] in the Supplementary Appendix).

### 2.6. Statistical Analysis

Data were summarized as means with standard deviations for continuous variables with normal distribution or medians with interquartile range (IQR) for those with nonnormal distribution. For categorical variables, values were reported as frequencies and proportions. Differences between groups were analyzed using Mann–Whitney test, Pearson *χ*^2^ test, or Fisher's exact test, as appropriate. The Kaplan-Meier method was used to estimate the cumulative rates of positive culture. All statistical analyses were performed using PASW (version 18.0, SPSS Inc., Chicago, IL, USA), and a two-sided *P* < 0.05 was considered significant.

## 3. Results

### 3.1. Patient Characteristics

Thirty-four patients received conventional regimen while 16 patients received intensified regimen in the study. None of the 50 patients tested positive for human immunodeficiency virus. The demographic characteristics of participants at baseline did not differ significantly between the treatment groups except age and body mass index. Five (14.7%) and 2 (12.5%) patients in the conventional regimen group and intensified group, respectively, were either unable to produce sputum or had negative sputum cultures and subsequently underwent bronchoscopy. A diagnosis in these cases was established via culture from bronchial washing or bronchoalveolar lavage. In the conventional regimen group, 28 patients (82.4%) had the nodular bronchiectatic form, 3 (8.8%) with the upper lobe cavitary form, and 3 (8.8%) with unclassifiable variants. In the intensified regimen group, 14 (87.5%) patients had the nodular bronchiectatic form, 1 (6.25%) with the upper lobe cavitary form, and 1 (6.25%) with unclassifiable variants (Table 1). Body mass index and age did differ significantly between the two groups.

### 3.2. Clinical and Microbiologic Responses

As shown in Table 2, response rates based on symptoms were 16 of 34 patients (47.1%) and 12 of 16 patients (75.0%) in the conventional regimen group and intensified regimen group, respectively, at 1 month (*P*=0.18), and 9 of 34 patients (26.5%) and 10 of 16 patients (62.5%), respectively, at 18 months (*P*=0.046).

**Figure 1:**
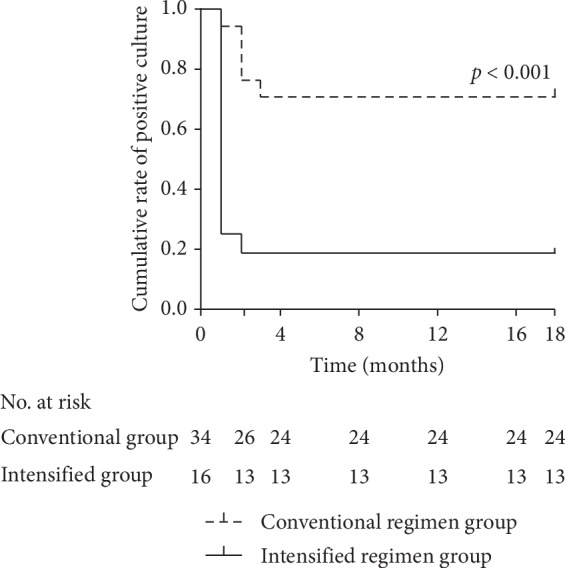
Cumulative rate of sputum positive culture in 50 patients with *M. abscessus* lung disease. The solid line shows the cumulative positive culture rate in the intensified regimen group while the dotted line shows cumulative positive culture rate in the conventional regimen group. Four cases in the intensified regimen group having recurrence are classified as fail in achieving sputum conversion.

More patients in the intensified regimen group than in the conventional regimen group had confirmed culture conversion at both 1 and 18 months: 2 of 34 patients (5.9%) and 12 of 16 patients (75%) in the two groups, respectively, at 1 month (*P* < 0.001), and 10 of 34 patients (29.4%) and 13 of 16 patients (81.3%), respectively, at 18 months (*P* < 0.001) (Figure 1).

In the intensified regimen group, the median time until sputum conversion was 1 month (IQR, 1-1 mo), compared with 2 months (IQR, 1-2 mo) in the conventional regimen group.

Among the 14 patients, who achieved initial sputum conversion after treatment in the conventional regimen group, 4 patients experienced a reversion of *M. abscessus* lung disease during the continuation phase. However, no patient in intensified regimen group experienced a reversion after sputum conversion.

### 3.3. Adverse Events

Compared with other antibiotics, adverse reactions associated with cefoxitin occurred frequently. Leukopenia (white blood cell counts, 2500/mm^3^) developed in 6 (37.5%) and 1 (2.9%) patients in the intensified regimen group and conventional regimen group, respectively. Drug-induced hepatotoxicity (alanine aminotransferase levels > 50 IU/L) occurred in 4 (25%) and 2 (5.9%) patients in intensified and conventional regimen groups, respectively (Table 3). Because of these adverse reactions, cefoxitin was discontinued in 6 (37.5%) patients after treatment for a median of 23 days (IQR, 21–25 d) in the intensified regimen group, compared with 1 patient in the conventional regimen group. After the discontinuation of cefoxitin, the above adverse reactions resolved completely. There were no complaints of vestibular dysfunction or hearing difficulties attributable to administration of amikacin.

Gastrointestinal symptoms (e.g., vomiting or nausea) associated with oral antibiotic usage occurred in 3 (8.8%) patients in the conventional regimen group and 2 (12.5%) patients in the intensified group. In the conventional regimen group, 2 patients were able to continue antibiotic therapy after reduction of the clarithromycin dosage (500 mg/d), whereas one patient required substitution of clarithromycin with azithromycin. Both of the patients in the intensified regimen group could tolerate drug after a short-term discontinuation.

Anemia associated with linezolid usage occurred in 1 patient in the intensified regimen group. Since the adverse event is classified as nonsevere, the patient completed treatment without discontinuation of linezolid. Arthralgia associated with fluoroquinolone occurred in 2 patients in the conventional regimen group and 1 patient in the intensified regimen group.

## 4. Discussion

*M. abscessus* is one of the leading causes of NTM disease globally, especially in East Asia. In Beijing Chest Hospital, *M. abscessus* lung disease accounts for 33.3% of the definite NTM lung disease cases [2]. A highly effective regimen to treat *M. abscessus* lung disease is still to be recommended. Although most *M. abscessus* strains were sensitive to cefoxitin, amikacin, and clarithomycin, treatment responses was underwhelming [3, 8]. Some scientists believe susceptibility of NTM could be overestimated in some occasions [11]. In 2010, our team initiated diagnosis and treatment of *M. abscessus* lung disease in China. *M. abscessus* lung disease cases were administered with a regimen based on a Korean experts' report. However, several medications are different from the previous report. Firstly, we decreased the dosage of cefoxitin since the drug was discontinued in more than half of patients due to adverse events, including leukopenia, hepatotoxicity, and thrombocytopenia, in patients as indicated in the previous report and since the incidence of *β*-lactam antibiotic-induced neutropenia increases when parenteral treatment is used in higher doses and extended beyond 2 weeks [12, 13]. Secondly, doxycycline was not administered due to poor sensitivity for *M. abscessus* isolates [7]. It is unlikely patients could benefit from doxycycline usage. The treatment regimen is not limited to Beijing Chest Hospital but also widely recommended in other mycobacterial referral centers in China.

Unfortunately, treatment responses of this conventional regimen are poor. Only less than one third of the patients achieved sputum conversion at 18 months after treatment. Despite initial conversion, 4 patients got sputum reversion after initial conversion. We speculated that *M. abscessus*, a rapidly growing bacterium, could be induced for drug resistance if an inadequate regimen in the initial phase is administered. In addition, inducible resistance to clarithromycin in *M. abscesssus* isolates is related to lack of efficacy of clarithromycin-containing antibiotic therapy against *M. abscessus* lung disease [7]. Combination with new antibiotics, which are effective to inhibit *M. abscessus* isolates, can probably improve treatment responses. Linezolid, to which 93% *M. abscessus* isolates are susceptible in Beijing Chest Hospital, seems to be a promising option [8]. Although new guidelines recommend an intensified regimen in the initial phase [14, 15], to the best of our knowledge, there have only been limited studies to investigate efficacy of such regimen. From Aug 2017, we initiated an intensified regimen to treat the disease. In contrast with the conventional regimen, the intensified regimen included a higher dosage of cefoxitin, linezolid, and azithromycin instead of clarithromycin. Sputum conversion is significantly higher and faster in the intensified regimen group when compared with the conventional regimen group.

As far as adverse events were concerned, leukopenia and hepatotoxicity are more common in the intensified regimen group. Since these events, related to cefoxitin, occurred in the first month, a close safety surveillance is necessary during that period. There was no significant difference for anemia between conventional and intensified regimen groups, although linezolid was administered in the latter. There was no significant difference for arthralgia, vomit, and nausea between two groups. Although 10 patients in the intensified regimen group had adverse events, only 1 patient had severe event which attributed to the modified regimen after close safety monitoring.

The present study has several limitations. Firstly, our study is a retrospective case study that was conduced at a single center. Although a prospective randomized controlled trial would be the proper way to solve the problem, it is difficult to initiate such a trial due to limited cases. Secondly, the number of patients was not enough to give a convincing answer for the problem. However, compared with conventional regimen, most likely a more aggressive treatment could improve treatment responses of *M. abscessus* lung disease.

In conclusion, an intensified regimen is helpful for a favorable outcome for *M. abscessus* lung disease. However, careful surveillance of complete blood count and hepatic function is necessary for safety surveillance.

## Figures and Tables

**Table 1:** Comparison of the clinical and radiographic characteristics of the patients with *Mycobacterium abscessus* lung disease receiving conventional and intensified regimen.

Characteristic	Conventional regimen (*n*=34)	Intensified regimen (*n*=16)	*P* value
Age (yr), median (IQR)	48 (39.8–55.3)	39.5 (36–43.8)	0.023
Sex, female	29 (85.3%)	15 (93.8%)	0.396
Body mass index (kg/m^2^), median (IQR)	23.95 (22.9–24.9)	24.7 (24.4–25.3)	0.018
Respiratory symptoms			
Cough	28 (82.4%)	14 (87.5%)	0.647
Sputum	28 (82.4%)	14 (87.5%)	0.647
Hemoptysis	6 (17.6%)	2 (12.5%)	0.647
Unable to produce sputum^*∗*^	5 (14.7%)	2 (12.5%)	0.836
Type of disease			
Nodular bronchiectatic form	28 (82.4%)	14 (87.5%)	0.892
Upper lobe cavitary form	3 (8.8%)	1 (6.3%)	
Unclassifiable variants	3 (8.8%)	1 (6.3%)	

^*∗*^Culture from bronchial washing or bronchoalveolar lavage.

**Table 2:** Clinical responses for *M. abscessus* lung disease.

	Conventional regimen (*n*=34)	Intensified regimen (*n*=16)	*P* value	Conventional regimen (*n*=34)	Intensified regimen (*n*=16)	*P* value
	Month 1			Month 18		
Improved	16	12	0.18	9	10	0.046
Unchanged	10	2		14	4	
Worsened	8	2		11	2	

**Table 3:** Adverse events according to the severity and treatment group.

Outcome	Conventional regimen (*n*=34)	Intensified regimen (*n*=16)	*P* value	Conventional regimen (*n*=34)	Intensified regimen (*n*=16)	*P* value
	No. with nonsevere event			No. with severe event		
WBC, decreased	1	6	0.001	0	1	0.14
ALT or SGPT, high	2	4	0.05	0	0	NA
Nausea	3	2	0.68	0	0	NA
Vomiting	3	2	0.68	0	0	
Creatinine, high	0	0	NA	0	0	NA
Hemoglobin, low (mmol/L)	0	1	0.14	0	0	NA
Arthralgia	1	1	0.58	0	0	NA

NA: not applicable.

## Data Availability

The data used to support the findings of this study are available from the corresponding author upon request.

## Supplementary Materials

Supplementary Materials Table S1 in the supplementary appendix shows adverse events were classified according to the Division of AIDS, National Institute of Allergy and Infectious Disease.
