# Supplementary material for: An Intensified Regimen Containing Linezolid Could Improve Treatment Response in Mycobacterium abscessus Lung Disease
Source: Biomed Res Int. 2019 Nov 19;2019:8631563. doi: 10.1155/2019/8631563 (PMC6885786; doi:10.1155/2019/8631563)
Supplement: Supplementary Materials — Table S1 in the supplementary appendix shows adverse events were classified according to the Division of AIDS, National Institute of Allergy and Infectious Disease. [file 8631563.f1.pdf]

Supplementary Appendix

Table S1

PARAMETER	GRADE 1 MILD	GRADE 2 MODERATE	GRADE 3 SEVERE	GRADE 4 POTENTIALLY LIFETHREATENING
WBC, Decreased (cells/mm3; cells/L)	2,000 to 2,499	1,500 to 1,999	1,000 to 1,499	< 1,000
ALT or SGPT, High	1.25 to < 2.5 x ULN	2.5 to < 5.0 x ULN	5.0 to < 10.0 x ULN	≥ 10.0 x ULN
Nausea	Transient (< 24 hours) or intermittent AND No or minimal interference with oral intake	Persistent nausea resulting in decreased oral intake for 24 to 48 hours	Persistent nausea resulting in minimal oral intake for > 48 hours OR Rehydration indicated (e.g., IV fluids)	Life-threatening consequences (e.g., hypotensive shock)
Vomiting	Transient or intermittent AND No or minimal interference with oral intake	Frequent episodes with no or mild dehydration	Persistent vomiting resulting in orthostatic hypotension OR Aggressive rehydration indicated (e.g., IV fluids)	Life-threatening consequences (e.g., hypotensive shock)
Creatinine, High	1.1 to 1.3 x ULN	> 1.3 to 1.8 x ULN OR Increase of > 0.3 mg/dL above baseline	> 1.8 to < 3.5 x ULN OR Increase of 1.5 to < 2.0 x above baseline	≥ 3.5 x ULN OR Increase of ≥ 2.0 x above baseline
Hemoglobin, Low (mmol/L)	male， 10.0 to 10.9 female, 9.5 to 10.4	male， 9.0 to < 10.0 female, 8.5 to < 9.5	male， 7.0 to < 9.0 female, 6.5 to < 8.5	male， < 7.0 female, < 6.5
Arthralgia	Joint pain causing no or minimal interference with usual social & functional activities	Joint pain causing greater than minimal interference with usual social & functional activities	Joint pain causing inability to perform usual social & functional activities	Disabling joint pain causing inability to perform basic self-care functions
